# Pseudomonas Mendocina Bacteremia in a Hemodialysis Patient With a Central Venous Catheter

**DOI:** 10.7759/cureus.10853

**Published:** 2020-10-08

**Authors:** Madeline E Goldberg, Michelle Blyth, Ed Swiatlo

**Affiliations:** 1 Internal Medicine-Pediatrics, Tulane University School of Medicine, New Orleans, USA; 2 Infectious Diseases, Tulane University School of Medicine, New Orleans, USA; 3 Infectious Diseases, VA Medical Center, New Orleans, USA

**Keywords:** pseudomonas, psuedomonas mendocina, bacteremia, hemodialysis, esrd, central venous catheter

## Abstract

*Pseudomonas mendocina* is an uncommon pathogen in humans and there are no documented cases of infection associated with central venous catheters. Here we describe a 72-year-old man on hemodialysis who presented with a fever and was found to have *P. mendocina* bacteremia. The only obvious source of infection was the hemodialysis catheter. The isolate was susceptible to all antibiotics tested and he was successfully treated with ciprofloxacin and central venous catheter removal. Patients with chronic medical conditions and vascular devices are at risk for invasive infections with rare Pseudomonas species. As laboratory pathogen detection advances arise, it is possible that additional cases of *Pseudomonas mendocina *infections in humans will be identified. Our case provides one example of the successful treatment of *Pseudomonas mendocina* bacteremia in a 72-year-old man with a line-associated infection.

## Introduction

Hemodialysis catheters are common sources of infection including bacteremia, and they are often needed in patients with end-stage renal disease (ESRD) for renal replacement therapy. Two types of circulatory access most commonly used for renal replacement therapy include arteriovenous fistulas (AVF) and central venous catheters (CVC). CVCs are less desirable as there is a greater risk of infection, however, they are often the best option when vascular access is limited. The relative risk of hospitalization due to infection and subsequent death is two to three times greater for CVCs compared to AVFs [1]. Common organisms implicated in CVC infections include gram-positive cocci such as staphylococci (coagulase-negative as well as *Staphylococcus aureus*), enterococci, Enterobacteriaceae, and Candida species. In some cases, catheters can be salvaged with catheter exchange over wire or antibiotic lock therapy, however, in most cases of catheter-related bloodstream infection (CRBI) the Infectious Diseases Society of America (IDSA) recommends that the catheter should be removed [2].

## Case presentation

This report describes a 72-year-old man who presented to a local hospital with fever and rapid heart rate. Past medical history included: ESRD (on dialysis) caused by immunoglobulin A (IgA) nephropathy; atrial fibrillation (on apixaban); heart failure with reduced ejection fraction (ejection fraction 30-35%); obesity; and chronic venous stasis of bilateral lower extremities. After the incidental discovery of a fever (102.5 °F) by the patient’s home health nurse, the patient was brought to the emergency department (ED). At the ED, patient denied infectious symptoms such as cough, shortness of breath, dysuria, or skin lesions with purulent drainage. Physical exam was notable for chronically edematous lower extremities of equal size with clear weeping. No infected ulcers or areas of erythema and tenderness were noted. Catheter site (right internal jugular vein) was non-tender, non-erythematous, and non-edematous.

The patient was found to have elevated white blood cell (24.33 cells/uL, normal reference values 4.8 - 10.8 cells/uL) and lactic acid (3.9 mmol/L, normal reference values 0.5 - 2.2 mmol/L); in addition, an electrocardiogram demonstrated atrial fibrillation with rapid ventricular response. Chest x-ray showed no consolidations and his urine culture had no growth. Blood cultures were obtained from peripheral sites and the patient was given one dose of vancomycin. The patient was transferred to our hospital and was admitted to the intensive care unit (ICU) for severe sepsis and atrial fibrillation with rapid ventricular rate necessitating an amiodarone drip. Antibiotic coverage was broadened to vancomycin and piperacillin/tazobactam and repeat blood cultures were drawn from peripheral sites as well as the CVC.

Following admission, he was quickly weaned from amiodarone and transferred to the general medicine floor. On day two of admission, peripheral blood cultures from the outside hospital, drawn prior to receiving antibiotics, grew gram-negative rods and vancomycin was discontinued. On day three of admission, these gram-negative rods were identified as *Pseudomonas mendocina* using matrix-assisted laser desorption/ionization-time of flight (MALDI-TOF). The medicine team consulted nephrology, infectious disease, and vascular surgery and all were in agreement that the CVC must be removed. On day four of admission, in vitro susceptibility testing results using microbroth dilution (MicroScan, Beckman Coulter, Inc., Brea, California, USA) were completed. There are no established breakpoints for in vitro susceptibility testing of *P. mendocina *but susceptibility testing of agents commonly used for other Pseudomonas species suggest that this isolate was susceptible to these antibiotics (Table 1).

**Table 1:** P. mendocina Antibiotic Susceptibility ^a^presumptive, as there are no established standards for in vitro susceptibility testing of *P. mendocina*.

Antibiotic	Susceptibility^a^	Minimal Inhibitory Concentration (MIC, micrograms per milliliter)
Amikacin	Susceptible	<8
Tobramycin	Susceptible	<2
Gentamicin	Susceptible	<1
Meropenem	Susceptible	<1
Ciprofloxacin	Susceptible	<0.5

Based on testing which showed a minimum inhibitory concentration (MIC) <0.5 for ciprofloxacin, antibiotic therapy was de-escalated to ciprofloxacin monotherapy. On day five of admission, the CVC was removed for a 48-hour line-free period. Oral ciprofloxacin was continued throughout this time and a new dialysis catheter (right chest wall trialysis catheter) was inserted on day eight of admission. Patient was discharged that same day after dialysis and completed oral ciprofloxacin at home for a total of 14 days. Patient was discharged home with home health services and with follow-up appointments with primary care, vascular surgery, and nephrology. Blood cultures collected at our facility remained negative throughout his hospitalization. 

## Discussion

*P. mendocina*, a gram-negative, non-fermentative bacillus, was discovered in 1970 in Mendoza, Argentina from soil and water [3]. It is an uncommon pathogen in humans. Since its identification, there have been 14 reported cases of *P. mendocina* infections in humans. Four cases presented with meningitis, five with endocarditis, and two with bacteremia. The remaining cases included peritonitis, septic arthritis, and spondylodiscitis (Table 2). The present case is the fifteenth case of *P. mendocina* infection reported globally and the third case in the United States. To our knowledge, this is the first reported case of *P. mendocina* CVC line-associated infection.

**Table 2:** P. Mendocina Cases

Publication Year	Location	Description
1992	Argentina	63-year-old man with prosthetic aortic valve with native mitral valve endocarditis [6].
2001	Denmark	28-year-old female with situs inversus with native tricuspid valve endocarditis [8].
2005	Taiwan	65-year-old man with alcohol hepatitis and chronic kidney disease with spondylodiscitis [7].
2007	Turkey	36-year-old man with native mitral valve endocarditis [9].
2011	France	79-year-old female with atrial fibrillation with native aortic valve endocarditis [10].
2011	Israel	31-year-old man with bacteremia [5].
2013	Singapore	34-year-old man with motor vehicle accident and foot wound infection leading to septic arthritis [11].
2016	United States	57-year old man with chronic alcohol use with native mitral valve endocarditis [12].
2017	Portugal	22-year-old man with peritoneal dialysis with peritonitis [13].
2018	Taiwan	55-year-old man with diabetes with meningitis [14].
2018	Taiwan	66-year-old female with external ventricular drain with meningitis [14].
2018	Taiwan	79-year-old man with chronic obstructive pulmonary disease with meningitis [14].
2018	Taiwan	78-year-old female with meningitis [14].
2019	United States	63-year-old man with human immunodeficiency virus/acquired immunodeficiency syndrome with bacteremia [4].
This study	United Sates	72-year-old man with end-stage renal disease on hemodialysis with bacteremia.

Each case of *P. mendocina* has unique details of presentation and treatment. In 2019, a case of *P. mendocina *bacteremia was reported in a patient with HIV who presented with fever, elevated lactate, and elevated white blood cell count. This patient was successfully treated with ceftazidime [4]. Another case of bacteremia occurred in a young man with *P. mendocina* bacteremia that was presumed to have been acquired from his pet bird’s drinking water [5]. A case of *P. mendocina* endocarditis occurred in a florist who the authors surmised acquired the bacterium from a cut contaminated by soil leading to bacteremia and hematogenous spread to the heart [6]. Lastly, *P. mendocina* has caused localized infection in the spine likely by hematogenous spread from a break in the skin and exposure to contaminated water or soil. This patient improved with source control and cefepime followed by oral ciprofloxacin [7].

Unfortunately, we are unable to definitively confirm the CVC as the source of infection in the present case, as the catheter was grossly contaminated during removal and thus the tip was not sent for culture. We can surmise that this was the most likely source of infection, as the physical exam did not suggest any other source of infection, and no other signs or symptoms were reported consistent with another infection. We believe at some point this patient's catheter site was exposed to contaminated soil or water which led to his infection.

Although there have been only 15 documented cases of *P. mendocina* infection in humans, it is possible that this number will rise as diagnostic advances such as mass spectrometry, polymerase chain reaction (PCR), and next-generation sequencing become widely deployed. These molecular assays allow the characterization of bacteria at a fine level of detail not possible with earlier phenotypic methods. This case adds to the literature as it presents an uncommon Pseudomonas species and catheter-associated infection and will help inform clinical approaches to *P. mendocina* infection in humans.

## Conclusions

*P. mendocina* is a gram-negative rod that has been isolated from environmental sources such as water and soil. It is seldom recovered from humans. This is the 15th case of *P. mendocina* infection in humans and is the first documented CVC line-associated infection. This case adds to the current literature by describing *P. mendocina* bacteremia in a patient with sepsis and the most likely source of infection was the CVC used for dialysis. Although not ideal, many patients require CVC and when patients develop line-associated catheter infections with Pseudomonas species, the IDSA currently recommends the removal of the catheter. This patient was successfully treated with catheter removal, followed by a 48-hour line-free period, replacement of a new catheter, and a 14-day course of oral ciprofloxacin based on in vitro susceptibility testing.

## References

[1] Dhingra RK, Young EW, Hulbert-Shearon TE, Leavey SF, Port FK (2001). Type of vascular access and mortality in U.S. hemodialysis patients. Kidney Int.

[2] Mermel LA, Allon M, Bouza E (2009). Clinical practice guidelines for the diagnosis and management of intravascular catheter-related infection: 2009 update by the Infectious Diseases Society of America. Clin Infect Dis.

[3] Palleroni NJ, Doudoroff M, Stanier RY, Solánes RE, Mandel M (1970). Taxonomy of the aerobic pseudomonads: the properties of the Pseudomonas stutzeri group. J Gen Microbiol.

[4] Gani M, Rao S, Miller M, Scoular S (2019). Pseudomonas mendocina bacteremia: a case study and review of literature. Am J Case Rep.

[5] Nseir W, Taha H, Abid A, Khateeb J (2011). Pseudomonas mendocina sepsis in a healthy man. Isr Med Assoc J.

[6] Aragone MR, Maurizi DM, Clara LO, Navarro Estrada JL, Ascione A (1992). Pseudomonas mendocina, an environmental bacterium isolated from a patient with human infective endocarditis. J Clin Microbiol.

[7] Chi CY, Lai CH, Fung CP, Wang JH (2005). Pseudomonas mendocina spondylodiscitis: a case report and literature review. Scand J Infect Dis.

[8] Johansen HK, Kjeldsen K, Høiby N (2001). Pseudomonas mendocina as a cause of chronic infective endocarditis in a patient with situs inversus. Clin Microbiol Infect.

[9] Mert A, Yilmaz M, Ozaras R, Kocak F, Dagsali S (2007). Native valve endocarditis due to Pseudomonas mendocina in a patient with mental retardation and a review of literature. Scand J Infect Dis.

[10] Suel P, Martin P, Berthelot G, Robaday S, Etienne M, Chibani A (2011). A case of Pseudomonas mendocina endocarditis. Med Mal Infect.

[11] Chiu LQ, Wang W (2013). A case of unusual Gram-negative bacilli septic arthritis in an immunocompetent patient. Singapore Med J.

[12] Rapsinski GJ, Makadia J, Bhanot N, Min Z (2016). Pseudomonas mendocina native valve infective endocarditis: a case report. J Med Case Rep.

[13] Jerónimo TM, Guedes AM, Stieglmair S, Guerreiro R, Laranjo C, Bernardo I, Neves PL (2017). Pseudomonas mendocina: the first case of peritonitis on peritoneal dialysis. Nefrologia.

[14] Huang CR, Lien CY, Tsai WC (2018). The clinical characteristics of adult bacterial meningitis caused by non-Pseudomonas (Ps.) aeruginosa Pseudomonas species: a clinical comparison with Ps. aeruginosa meningitis. Kaohsiung J Med Sci.

